# Optimal timing of chest radiograph following chest tube removal in detecting recurrent pneumothorax: analysis of 163 patients with thoracic stab wounds at a major trauma centre in South Africa

**DOI:** 10.1308/rcsann.2025.0018

**Published:** 2025-06-19

**Authors:** V Kong, J Ko, C Cheung, H Lee, R He, J Wang, D Clarke

**Affiliations:** ^1^ University of Auckland, Auckland, New Zealand; ^2^ Auckland City Hospital, Auckland, New Zealand; ^3^ University of Kwa-Zulu Natal, Durban, South Africa; ^4^ Chris Hani Baragwanath Hospital, Johannesburg, South Africa; ^5^ University of the Witwatersrand, Johannesburg, South Africa; ^6^ Lyell McEwin Hospital, Adelaide, Australia

**Keywords:** Wounds, Stab, Pneumothorax, X-rays

## Abstract

**Introduction:**

Routine chest radiograph (CXR) following chest tube removal is a common practice, yet the optimal timing of CXR in detecting recurrent pneumothorax (RPTX) remains unknown. This study reviewed the incidence of RPTX and its relationship to the timing of the detection of CXR.

**Methods:**

A prospective study was conducted over a 24-month period on patients with thoracic stab wounds who underwent CXR following chest tube removal at a major trauma centre in South Africa.

**Results:**

One hundred and sixty-three patients were included (91% male, mean age: 25 years). Eleven patients (7%) had RPTX, one (9%) of whom required reinsertion of a chest tube. No patients were readmitted following discharge. The timing of the CXR was: <2h (11%), 2–4h (21%), 4–6h (28%), 6–8h (31%) and >8h (9%). Of the 11 RPTX, 55% were detected on CXR at <2h, 36% at 2–4h, 9% at 4–6h, 0% at 6–8h (0%) and 0% at >8h. All RPTX were detected within <6h of chest tube removal. There was no re-presentation of any patients following discharge.

**Conclusion:**

RPTX following chest tube removal is uncommon, and the need for reintervention is low. All patients with RPTX were detected on CXR obtained within 6h of removal. It would appear that routinely delaying CXR anytime beyond 6 hours is unnecessary.

## Introduction

Tube thoracostomy (TT) is a commonly performed procedure in the management of thoracic trauma, and the vast majority of penetrating thoracic trauma secondary to a stab wound can be managed by TT alone.^1–6^ Obtaining a post-chest tube removal chest radiograph (CXR) is common practice because recurrent pneumothorax (RPTX) can develop, which may necessitate reinsertion of the chest tube.^4,5^ RPTX following chest tube removal is a well-known phenomenon, with a reported rate in the literature of up to 12%.^4–11^ However, the need for repeat chest tube insertion for RPTX is usually uncommon.^10,11^ An area of controversy in the management of TT is the optimal timing in obtaining CXR following chest tube removal.^6,7^ This practice is heterogeneous, with various authors recommending a timing ranging from 1 to 24h post chest tube removal.^6,7^ Theoretically, a delay between chest tube removal and the post-removal CXR may detect a slowly developing RPTX. Other authors opine that any RPTX which may develop is usually related to the removal technique and, thus, should be detectable immediately without a prescribed waiting period.

Furthermore, the optimal timing of CXR has never been formally subjected to detailed studies in non-ventilated trauma patients.^10^ Delay in obtaining a CXR may postpone patient discharge and thus is especially relevant in a resource-contained environment. This study aimed to review the incidence of RPTX in patients with thoracic stab wounds following chest tube removal and to interrogate the correlation between the timing of post-removal CXR and the detection of RPTX.

## Methods

### Clinical setting

This study was undertaken in the Pietermaritzburg Metropolitan Trauma Service (PMTS), Pietermaritzburg, South Africa, over a 24-month period from January 2012 to December 2013. This study was a retrospective analysis of a prospectively collected data set. The PMTS provides definitive trauma care for the western part of the KwaZulu Natal (KZN) province and the city of Pietermaritzburg. It is one of the largest academic trauma centres in the province, covering a catchment population of more than 3 million. Our centre manages more than 5,000 admissions annually, with >50% of admissions secondary to penetrating injuries. Ethics approval for this study and for the maintenance of trauma registry data was formally endorsed by the Biomedical Research Ethics Committee (BREC) of the University of KwaZulu Natal (UKZN) (Reference number: BE 207/09).

### Inclusion criteria

All patients who sustained isolated thoracic stab wounds and required TT for pneumothorax were reviewed. Only those who had a TT performed in the trauma resuscitation bay and who did not subsequently require a laparotomy or thoracotomy were included. All patients with either a haemopneumothorax or haemothorax were excluded. All patients who went on to require mechanical ventilation were excluded, as were patients who needed more than one chest tube at the initial assessment. All patients who had their chest tube removed and a post-removal CXR were included in the study.

### Management protocol

The duty trauma resident assesses all patients who sustain a thoracic stab wound, and if indicated, a chest tube is inserted in the unit prior to moving the patient out of the trauma bay. The insertion technique is standardised, and we follow the recommendations of the Advanced Trauma Life Support (ATLS).^1^ TT is performed under local anaesthesia. A skin incision is made anterior to the mid-axillary line in the fifth intercostal space, within the triangle of safety. Blunt dissection of the intercostal space with forceps is followed by inserting a finger through the intercostal muscle into the pleural space. Following this finger sweep, a chest tube is inserted into the pleural cavity and connected to an underwater bottle filled with 500ml of saline.

The chest tube is removed once the following clinical criteria are met: the patient must be clinically stable, not in distress and have normal respiratory parameters; the underwater bottle fluid level must not swing on respiration; and the TT must not drain more than 50ml daily. A pre-removal CXR is obtained to confirm complete expansion of the lung. During the study period of January 2012 to December 2013, all chest tubes were removed using the end-of-expiration technique. All chest tube removals were performed by the primary author (VK) or under the primary author’s direct supervision. All chest tubes were removed using a two-person technique. Once the chest tube is removed by the clinician, an occlusive dressing is placed immediately over the wound site (by the second operator) to provide a seal. All patients are then sent for a routine post-removal CXR.

### The study

All patients who underwent CXR following chest tube removal were reviewed. A CXR was requested immediately following the removal of a chest tube. At the time of the study, there was no departmental policy stipulating the exact timing for CXR. Therefore, the CXR was taken by the radiographer when they were available. The time from chest tube removal to the CXR was reviewed and categorised as: <2h, 2–4h, 4–6h, 6–8h and >8h. All CXRs were reviewed by the primary author (VK) and the duty consultant trauma surgeon. All patients without an RPTX on CXR are discharged immediately and advised to return to the hospital if they become symptomatic. Patients with an RPTX on CXR are observed for a further 24h. A new TT is inserted based on the patient’s clinical status during the period of in-hospital observation.

### Statistical analysis

All data were extracted onto a Microsoft Excel© spreadsheet and imported into SPSS version 27 (IBM Corp., released 2010; IBM SPSS Statistics for Windows, version 19.0) for processing and analysis.

## Results

### Overview

During the 24-month study period, a total of 163 patients who sustained a pneumothorax secondary to an isolated thoracic stab wound and required TT were included. Ninety-one per cent were male (148 of 163), and the mean age was 25 years. All 163 were patients from South Africa. Eleven patients (7%) had RPTX demonstrated on post-removal CXR. One of these 11 patients (9%) required reinsertion of a chest tube. No patients were readmitted following discharge.

### Timing of CXR

The timing of CXR post chest tube removal for the 163 patients is summarised in Table 1.

**Table 1:** Timing of chest radiograph following chest tube removal in 163 patients

Time	Total number (*N* = 163)	%
<2h	18	11
2–4h	35	21
4–6h	46	28
6–8h	50	31
>8h	14	9

### Recurrent pneumothorax

**Figure 1:**
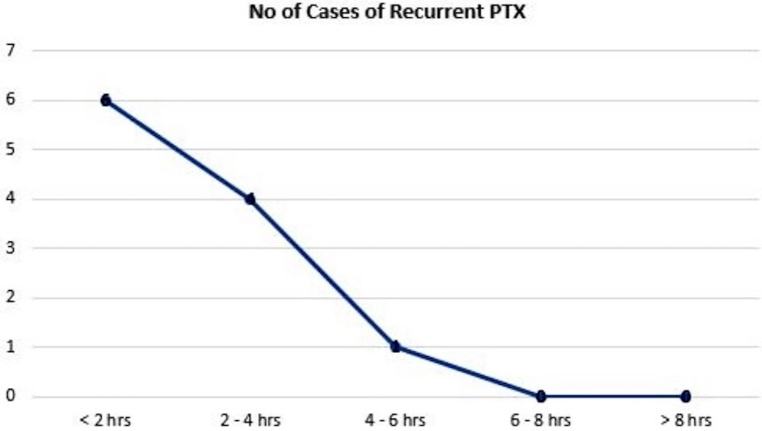
Graphical representation of number of recurrent pneumothorax detected in relation to timing of chest radiograph

Table 2 summarises the number of patients who had RPTX detected in relation to the timing of the CXR following chest tube removal. Figure 1 summarises the data in graph form. Of the 11 patients with RPTX, 1 experienced clinical deterioration and required reinsertion of a chest tube. The CXR was performed less than 2h post chest tube removal. The patient’s occlusive dressing was noted to be completely non-adherent to the chest wall. Ninety-one per cent of all RPTXs were detected on CXR less than 4h post TT removal and 100% on CXR less than 6h post TT removal. No RPTXs were noted on CXR beyond 6h following chest tube removal.

**Table 2:** Recurrent pneumothorax categorised by the timing of chest radiograph

Time	Recurrent pneumothorax (*N* = 11)	%
<2h	6	55
2–4h	4	36
4–6h	1	9
6–8h	0	0
>8h	0	0

## Discussion

TT is commonly performed in the management of penetrating thoracic trauma, and the standardised insertion technique has been extensively taught as part of ATLS.^1^ The role and timing of CXR in the process of insertion and removal have been much debated over the past five decades.^7–10^ The points of controversy include the actual need for a routine post-insertion CXR as well as the need for and timing of the post-removal CXR.^7,8^ Many trauma centres continue to obtain a routine CXR following chest tube removal. This is not unreasonable because there is a small but definite incidence of RPTX following chest tube removal, which may require repeat intervention.^5,11^ This is one of the primary justifications for the practice of routine CXR following chest tube removal. The incidence of RPTX in our study (7%) was consistent with that reported in the literature.^4,6–10^ Repeat intervention for RPTX was relatively rare, with only one patient in our series requiring such intervention. This was almost certainly related to a technical error. Other factors may potentially influence the development of RPTX. Studies from both the United States and our own institution in South Africa have conclusively demonstrated that the timing of TT removal in relation to the respiratory cycle does not appear to influence the development of RPTX.^4,11^

The optimal timing of post-removal CXR, however, remains highly controversial.^4,7,8,10,11^ The theoretical advantage of waiting for a specific time is based on the assumption that a certain period is required to elapse prior to the development of an RPTX. Thus, a CXR performed immediately post removal of the chest tube may miss the RPTX, which develops over the next few hours. The only study so far in the English literature that directly addresses the question of the timing of post-removal CXR was by Pizano *et al*. In their study of 75 mechanically ventilated trauma patients, CXR obtained within 1h following chest tube removal appeared to be sufficient.^10^ For non-ventilated patients, the timing reported in the literature varies between 1 and 24h, with some authors advocating an interval repeat CXR up to 24h post removal.^7–9,10–17^

Our current study demonstrated that more than 50% of patients with RPTX were detected on CXR performed less than 2h post chest tube removal, and 90% were detected within 4h. Virtually all RPTXs were detected within 6h post chest tube removal. This is consistent with our clinical experience in that delaying CXR beyond 6h post chest tube removal has little benefit and merely delays patient discharge. A significant number of patients in our study had their post-chest tube removal CXR after 6h. This reflects the busy nature of our under-resourced institution, which, at times, has managed more than 150 trauma patients over a 24-h period. However, obtaining CXR earlier than 6h appears to be a safe and pragmatic strategy, which may facilitate earlier patient discharge. It should be noted that no patient who was discharged without RPTX returned to the hospital. To our knowledge, our study is the only one so far that specifically addresses the question of optimal timing of obtaining CXR following chest tube removal in the context of PTX secondary to thoracic stab wounds.

### Study limitations

One of the major limitations of this study is the lack of randomisation regarding the timing of CXR. Furthermore, the actual removal technique itself, although consistent (and supervised by a single clinician), is specific to our institution and the practice may differ elsewhere. Another limitation is related to the criteria for initiating chest tube removal. This practice varies widely between individual institutions. At our trauma centre, our criteria are conservative. It was not possible to ascertain whether those who had CXR less than 6h following chest tube removal subsequently went on to develop RPTX that was not detected on the initial CXR. None of these patients re-presented following their discharge. However, most studies would suggest that if RPTX is significant, it will manifest clinically. It would appear that once the chest tube is removed, CXR can be obtained within 4h to detect RPTX. Waiting beyond 6h to obtain CXR appears to be unnecessary. One of the major limitations of this study is that it is based on a single centre’s experience. It would be suitable to a multicentre trial for definitive conclusion in the future.

## Conclusions

RPTX following chest tube removal is uncommon and the need for reintervention is low. All patients who had RPTX were detected on CXR obtained within 6h of removal. It would appear that routinely delaying CXR anytime beyond 6h is unnecessary and merely delays patient discharge. A multicentre trial would be beneficial for a definite conclusion.
